# Cardiac scoring systems, coronary artery disease and major adverse cardiovascular events: A scoping review

**DOI:** 10.4102/safp.v65i1.5683

**Published:** 2023-08-10

**Authors:** Preesha Premsagar, Colleen Aldous, Tonya Esterhuizen

**Affiliations:** 1 Department of Internal Medicine, Nelson R. Mandela School of Medicine, University of KwaZulu-Natal, Durban, South Africa; 2 Department of Clinical Medicine, Nelson R. Mandela School of Medicine, University of KwaZulu-Natal, Durban, South Africa; 3 Division of Epidemiology/Biostatistics, Department of Global Health, Faculty of Medical and Health Sciences, Stellenbosch University, Stellenbosch, South Africa

**Keywords:** scoping review, coronary artery disease CAD, cardiac scoring systems, major adverse cardiovascular events MACE

## Abstract

**Background:**

In 2019, the World Health Organization (WHO) declared coronary artery disease (CAD) as the leading cause of death globally for the last 20 years. Early screening and detection (primary prevention) and intervention (secondary prevention) are necessary to curb CAD and major adverse cardiovascular event (MACE) prevalence. A scoping review to assess the current literature on using cardiac scoring systems to predict CAD and MACE was performed.

**Methods:**

The research question ‘What is the literature on using cardiac scoring systems to predict CAD and MACE?’ was addressed. The updated Arksey and O’Malley and the Preferred Reporting Items for Systematic reviews and Meta-Analyses Extension for Scoping Reviews methodologies were used. The search terms ‘coronary artery disease’ and ‘cardiac scoring systems’ and ‘major adverse cardiovascular events’ were used in the Boolean search on PubMed, ScienceDirect, MedLine and Cochrane Library.

**Results:**

The final list consisted of 19 published English results after the year 2000. There were six results without participants (four clinical guidelines, one review article and one ongoing clinical trial). Scoring systems were cardiovascular risk estimation systems focusing on the primary prevention of CAD; MACE was discussed but not scored. There were 13 robust results published from completed multinational clinical trials with participants. These results focused on a scoring system for the secondary prevention of CAD and MACE.

**Conclusion:**

Scoring systems remain an objective method for primary and secondary prevention of CAD and MACE.

**Contribution:**

Scoring systems may be helpful with clinical uncertainty or to standardise patient results for comparison in research.

## Introduction

In 2020, the World Health Organization (WHO) declared coronary artery disease (CAD) to be the leading cause of death globally for last 20 years. Moreover, CAD had escalated to 9 million cases in 2019, representing 16% of total deaths worldwide.^1^ Reported statistics in 2022 suggest that the prevalence of CAD and related deaths had further increased due to severe acute respiratory syndrome coronavirus 2 (SARS-CoV-2), with many overall under-reported cases.^2^ The impact of CAD and, subsequently, of major adverse cardiovascular events (MACE) has been explored in every aspect, including, but not limited to, quality of life, life expectancy, financial implications, overall health economic effects and mortality. Major adverse cardiovascular event incurs high costs in medical care for patients, their families and society.^3,4,5,6,7^ Evidence suggests a growing prevalence of CAD annually, causing a substantial overall disease burden measured in disability-adjusted life-years lost (DALY). Coronary artery disease and cerebrovascular accidents (CVA) result in substantial long-term morbidity and are confirmed to be the leading causes of overall disease burden as measured in DALYs.^7^

Early screening and detection (primary prevention), and intervention (secondary prevention)^8^ remain the most reasonable method to curb future CAD and MACE prevalence. The literature on scoring systems used for primary and secondary prevention for CAD and MACE is evaluated in this scoping review.

### Research question and objective

Our research question was, ‘What is the literature on using cardiac scoring systems to predict CAD and MACE?’ The objective of this scoping review was to map the literature on the use of cardiac scoring systems to predict CAD and MACE.

## Method

A literature review following the updated Arksey and O’Malley scoping review methodology was conducted^9^ and further refined by the Preferred Reporting Items for Systematic reviews and Meta-Analyses Extension for Scoping Reviews (PRISMA-ScR) using the checklist.^10,11^ Criteria for eligibility were results published from the year 2000 onwards, with full text available in English, that were relevant to the research question. The Arskey and O’Malley scoping review guidelines do not require non-English or previous-century literature to be removed; it remains an option. Literature before 2000 and non-English literature were removed to ensure that the search is relevant, contemporary and contextually understandable. A protocol was not designed for the scoping review specifically but was designed and accepted for a dissertation that followed the scoping review (Registration at the University of KwaZulu-Natal, BE 513/17).

A literature search was conducted on 01 October 2022. The search terms ‘coronary artery disease’ and ‘cardiac scoring systems’ and ‘major adverse cardiovascular events’ were used in the Boolean search on PubMed, ScienceDirect, MedLine and Cochrane Library databases. These databases were used because they are the most relevant concerning the subject matter and research question. Selected results included research articles, review articles, clinical guidelines, meta-analyses and randomised clinical trials (RCTs). Protocols and registries from RCTs were also included as part of the scoping review. Results of all methods (qualitative, quantitative and mixed analyses) were included. Data charting were done by a single reviewer and confirmed by an information technologist for consistency and accuracy.

For the purpose of this scoping review:

- Cardiac scoring systems included all numerically evaluated systems for cardiovascular risk estimation systems, image-based systems and investigation result scores that were used to assess current disease severity or evaluate future risk.
- Coronary artery disease referred to a confirmed acute coronary event such as a myocardial infarction or significant luminal stenosis ≥ 50% of in one or more major coronary vessel(s) on angiography.
- Primary prevention referred to medical and lifestyle treatment to screen, detect and prevent CAD.^8^
- Any other conditions not yet confirmed were considered suspected CAD for investigation.
- The definition of MACE was taken as a cardiac event (including confirming CAD in a patient who is already on primary prevention) or anyone other adverse complication of CAD, such as re-infarction, stent blocking and heart failure.
- Secondary prevention referred to interventional, medical and/or lifestyle treatment to prevent MACE.^8^

The results extracted from the databases were transferred to Endnote version 20.0. This included all the results obtained from Boolean search terms applied to the databases. Unavailable results were manually found through the University of KwaZulu-Natal library. The inclusion and exclusion criteria were methodically applied for charting (Table 1) and sifted by relevance, usefulness, processes and context to the overall objective. Abstracts, and full articles, whenever available, were read for a suitable selection in the final results. Every effort was made to ensure that the approach was uniform and consistent in the final selection, such as carefully adhering to the inclusion and exclusion criteria and removing grey literature that was not fully clear or transparent. The evidence gathered was charted.

**TABLE 1:** Inclusion and exclusion criteria of results extracted from the databases.

Inclusion criteria	Exclusion criteria
The timeline was set from 2000 up to 2022	Studies before year 2000
American English and United Kingdom English results	Non-English results with no option for a complete English version
Published results, consisting of research articles, review articles, clinical guidelines and clinical trial protocols and registries, meta-analyses. Studies of various methods were including qualitative, quantitative and mixed analyses	Grey literature results including unpublished, pre-print, non-peer reviewed results
Results had to be related to the research question	Results on drug trials, cardiac devices trials, cross-over with other topics, niche and/or specific therapeutic area or other results that are not related to the research question

### Ethical considerations

Ethical clearance to conduct this study was obtained from the Biomedical Research Ethics Committee of the University of KwaZulu-Natal (reference no. BE513/17).

## Results

**FIGURE 1:**
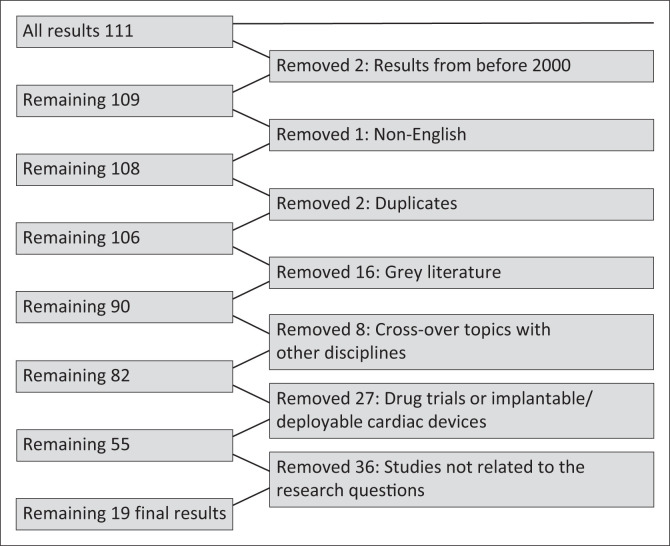
A chart of online and manual search results of the databases.

There were 111 results returned that matched the search terms on the databases through online and manual searching (Figure 1 and Table 2). These results were transferred to EndNote 20.0.

**TABLE 2:** All results of online and manual searches that matched the search terms on the databases (*N* = 111).

Type of paper	ScienceDirect	PubMed	MedLine	Cochrane Library	Total
Research article	21	5	0	45	71
Conference abstract	0	0	0	13	13
Review article	1	0	6	2	9
Clinical guidelines	0	0	8	0	8
Clinical trial protocol or registry	0	0	0	6	6
Letter to editor	0	0	1	1	2
Conference paper	0	0	0	1	1
Periodical	0	0	1	0	1

**Total**	**22**	**5**	**16**	**68**	**111**

There were two results from before 2000 (1983 and 1996), one non-English result (Korean) and two duplicates removed. There were 106 results remaining. After that, 16 grey area results (unpublished, pre-print, not peer-reviewed) were removed. The Arksey and O’Malley scoping review guidelines do not require the removal of grey literature from the search. However, grey literature was removed in this scoping review to be uniform and consistent; literature that was not clear and transparent, inclusion and/or exclusion criteria could not be confidently applied, or sources, authors and institutions that were not easily identified were removed. Thereafter, 90 results remained. Eight results were removed from cross-over topics with other disciplines (such as oncology, transplantation medicine, gestation, etc.), and 82 remained.

There were 27 results removed because they were on specific interventional RCT protocols, registries or studies, drug trials (such as immune-suppressants, dietary supplements and investigational molecules) or implantable/deployable cardiac devices (such as stents and surgical devices), and 55 results remained. Lastly, a further 37 results were removed because, although they were selected in the search terms on the databases, upon reading the articles, these results studied very niche areas (for example, intraoperative risk scoring for cardiac complications) and therefore were not related to the research question.

### General audit

The final set of results that best matched the research questions were 19 results (19/111 is 18% of the original result search with search terms) (Figure 1 and Table 3). These 19 final results ranged from 2007 to 2022 and contained 13 research articles, four clinical guidelines, one review article and one ongoing registered clinical trial (Table 3).

**TABLE 3:** Final results that best matched the research questions (*n* = 19).

No.	Type	References
1	Research article	Palmerini et al.^12^
2	Research article	Genereux et al.^13^
3	Research article	Dangas et al.^14^
4	Research article	Gershlick et al.^15^
5	Clinical guidelines	Scottish Intercollegiate Guidelines Network^16^
6	Research article	Taşolar et al.^17^
7	Review article	Skelly et al.^18^
8	Clinical guidelines	National Institute for Health and Care Excellence (NICE)^19^
9	Research article	Hirji et al.^20^
10	Research article	Yudi et al.^21^
11	Clinical guidelines	Scottish Intercollegiate Guidelines Network^22^
12	Research article	Redfors et al.^23^
13	Research article	Ferencik et al.^24^
14	Clinical guidelines	Scottish Intercollegiate Guidelines Network^25^
15	Research article	Hara et al.^26^
16	Research article	Takahashi et al.^27^
17	Research article	Sen et al.^28^
18	Research article	Takahashi et al.^29^
19	Clinical trial	Clinical trial iCorMicA^30^

Note: Please see the full reference list of the article, Premsagar P, Aldous C, Esterhuizen T. Cardiac scoring systems, coronary artery disease and major adverse cardiovascular events: A scoping review. S Afr Fam Pract. 2023;65(1), a5683. https://doi.org/10.4102/safp.v65i1.5683, for more information.

### Two types of results were distinguished

- Without patients. Focusing on primary prevention of CAD. Major adverse cardiovascular events discussed but not scored (*n* = 6).
- With patients. Focusing on secondary prevention of CAD and MACE (*n* = 13).

### Results without participants (*n* = 6)

Out of the 19 results, six had no participants; these were four clinical guidelines, one review article and one ongoing RCT. The RCT included in the final list of 19 was observational, did not involve a specific interventional drug or device, and had no listed patients on the search date.^30^ The guidelines and review article focused on scoring systems, which were also subjective questionnaires, to establish risk for CAD. Thereafter further tests were performed. Management was broadly discussed with a focus on treatment options for both CAD and other causes of chest pain. They were focused on cardiovascular risk estimation scoring systems for primary prevention and were dedicated to screening and detection of CAD in situations where the clinical evidence was non-specific and patients needed risk stratification. These results were captured in the Boolean Search because MACE was also discussed, but the scoring systems for MACE were not explored further. The sources of these clinical guidelines and review articles were from the United Kingdom or the United States. These six results were not included in the individual sources of evidence analysis because they were general guidelines on primary prevention and did not include actual patient participation.

### Results with participants (individual sources of evidence) (*n* = 13)

**FIGURE 2:**
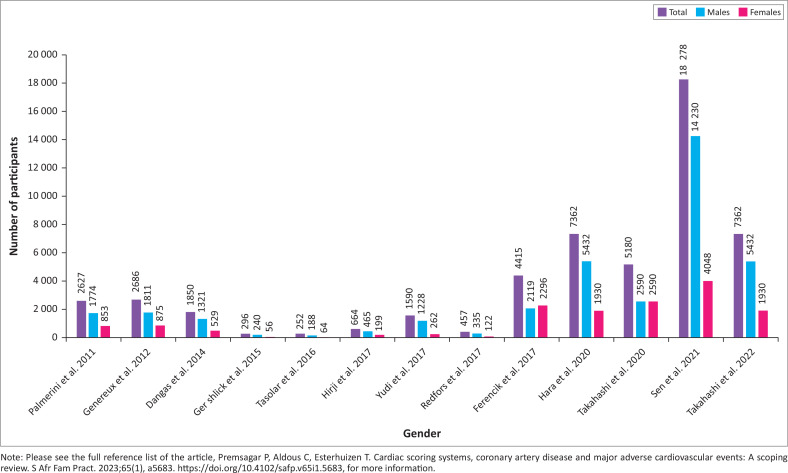
Number of participants for results in chronological order of publication (*n* = 13).

The 13 remaining results that were included in the individual sources of evidence analysis were the research articles with enrolled participants. Many studies were subgroups of more extensive registered clinical trials (National Clinical Trial [NCT] numbers provided). Three studies^26,27,29^ consisted of overlap from different clinical trials. Trials were multinational and were located at sites worldwide. The smallest and largest study had 296 and 18 278 participants, respectively. The studies showed an overall increase in the number of participants over the years (Figure 2), with more male participants.

Unlike the six results without participants, these 13 consisted of enrolled participants with established CAD at baseline, often having undergone intervention, such as bypass grafting. The scoring systems were more objective, and the SYNTAX score was the most frequently evaluated scoring system. The 13 results with participants focused technically on the inherent advantages and disadvantages of various forms of intervention in groups and subgroups of individuals. Major adverse cardiovascular events were measured over variable periods ranging from 1 to 5 years. The results measured the MACE endpoint, with all-cause mortality and morbidity as the primary outcome. The other individual MACE endpoints were measured, which varied from study to study (Table 4). These studies were focused on scoring systems for secondary prevention (and tertiary) and therefore were dedicated to the intervention of CAD and MACE.

**TABLE 4:** Baseline and major adverse cardiovascular events of individual sources of evidence (*n* = 13).

No.	Result	Baseline assessed						MACE assessed							
		NSTEMI, STEMI	PCI	Stable outpatients and family history	Multivessel or complex disease	CABG	Diabetes on insulin	All-cause mortality/morbidity	Death	Acute coronary syndrome/MI	Revascularisation	Stroke	Heart failure	Thrombo-embolic disease	Arrhythmia
1	Palmerini et al.^12^	X	X	-	-	-	-	X	X	X	X	-	-	-	-
2	Genereux et al.^13^	-	X	-	-	-	-	X	X	X	X	-	-	-	-
3	Dangas et al.^14^	-	-	-	-	-	X	X	X	X	-	X	-	-	-
4	Gershlick et al.^15^	-	X	-	X	-	-	X	X	X	X	-	X	-	-
6	Taşolar et al.^17^	X	-	-	-	-	-	X	-	-	-	-	-	-	-
9	Hirji et al.^20^	X	-	-	-	-	-	X	X	-	X	-	-	-	-
10	Yudi et al.^21^	-	-	X	-	-	-	X	X	X	-	X	-	-	-
12	Redfors et al.^23^	X	-	-	-	X	-	X	-	X	X	X	-	-	-
13	Ferencik et al.^24^	-	-	X	-	-	-	X	X	X	-	-	-	-	-
15	Hara et al.^26^	-	-	-	X	-	-	X	X	-	-	-	-	-	-
16	Takahashi et al.^27^	-	-	-	X	-	-	X	X	X	-	X	-	-	-
17	Sen et al.^28^	-	-	X	-	-	-	X	-	-	-	-	-	X	X
18	Takahashi et al.^29^	-	X	-	-	X	-	X	-	-	-	-	-	-	-

	**Total**	**4**	**4**	**3**	**3**	**2**	**1**	**13**	**9**	**8**	**5**	**4**	**1**	**1**	**1**

Note: Please see the full reference list of the article, Premsagar P, Aldous C, Esterhuizen T. Cardiac scoring systems, coronary artery disease and major adverse cardiovascular events: A scoping review. S Afr Fam Pract. 2023;65(1), a5683. https://doi.org/10.4102/safp.v65i1.5683, for more information.

NSTEMI, non-ST elevation myocardial infarction; STEMI, ST elevation myocardial infarction; PCI, percutaneous intervention; CABG, coronary artery bypass graft; MACE, major adverse cardiovascular events.

The baseline assessment of the 17 results included:

- 4 with NSTEMI/STEMI
- 4 with PCI
- 3 with multivessel/complex disease
- 3 with outpatients/family history
- 2 with CABG
- 1 with diabetes on insulin.

Individual MACE endpoints measured on results were: eight death, eight ACS, five revascularisations, four stroke, one heart failure, one thromboembolic disease and one arrhythmia (Table 4).

### Representation of the outcome of major adverse cardiovascular events from the individual sources of evidence

These 13 studies also presented their findings in various ways. Eight studies presented findings as a hazard ratio, four as a c-index, and one as a receiver operating characteristic (ROC) curve, with the area under the curve calculated. Groups were compared based on the baseline differences in risk factors (such as people with diabetes and people without diabetes) or treatment groups (such as PCI or bypass), and the scoring system was used to measure the MACE outcome (Table 5 and Table 6).

**TABLE 5:** Characteristics of data extracted (*n* = 19).

No	Reference	Trial paper	*N*	CAD baseline	Scoring systems	MACE	Patient participation
1	Palmerini et al.^12^	ACUITY	2 627	NSTEMI patients undergoing PCI	SYNTAX	1-year MACE follow-up (death, MI and target vessel revascularisation)	Yes
2	Genereux et al.^13^	No	2 686	Post PCI	SYNTAX	1-year MACE follow-up (death from any cause, MI or unplanned revascularisation)	Yes
3	Dangas et al.^14^	FREEDOM	1 850	Insulin-treated diabetes mellitus patients post CABG	SYNTAX	Death, stroke and MI	Yes
4	Gershlick et al.^15^	CvLPRIT	296	Multivessel disease undergoing primary PCI for STEMI	CASS	12-month follow-up MACE (all-cause death, recurrent MI, heart failure and ischaemia-driven revascularisation)	Yes
5	Scottish Intercollegiate Guidelines Network^16^	No	N/A	All chest pain	HEART	Short-term (30-day) long-term MACE	No
6	Taşolar et al.^17^	No	252	NSTEMI at baseline, with medical co-morbidities	CHA2DS2-VASc-HS, TIMI, GRACE and SYNTAX	CAD severity and complexity	Yes
7	Skelly et al.^18^	No	N/A	Pre-test likelihood based on chest pain characteristics and demographics	Calcium score	ACS, bypass and stenting	No
8	National Institute for Health and Care Excellence^19^	No	N/A	All chest pain	Pre-test score (history, examination, routine bloods and resting 12-lead ECG)	Risk of adverse events	No
9	Hirji et al.^20^	No	664	STEMI patients with conservatively managed non-IRA lesions	A 15-point risk score (risks in non-IRA, left ventricular ejection fraction, hypertension, heart failure and renal insufficiency)	5-year MACE follow-up (death, MI and urgent revascularisation)	Yes
10	Yudi et al.^21^	EXCEL	1 590	A family history of premature CAD	SYNTAX	5-year MACE follow-up (death, MI and stroke)	Yes
11	Scottish Intercollegiate Guidelines Network^22^	No	N/A	Typical angina and non-anginal chest pain	Framingham, Assign, JBS3 and Qrisk2	Risk of adverse events	No
12	Redfors et al.^23^	ACUITY	457	Patients with NSTEMI with CABG	SYNTAX	1-year all-cause death and MACE follow-up (MI, stroke and urgent revascularisation)	Yes
13	Ferencik et al.^24^	PROMISE	4 415	Stable outpatients with symptomatic chest pain	ASCVD	MACE (death, MI and unstable angina)	Yes
14	Scottish Intercollegiate Guidelines Network^25^	No	N/A	All chest pain	SYNTAX, NYHA, SAQ, Calcium score, Karnofsky Performance Scale and Duke Activity Score	5-year MACE	No
15	Hara et al.^26^	BEST, FREEDOM, EXCEL	7 362	Patients with 3VD and/or LMCAD	SYNTAX score II	5-year death	Yes
16	Takahashi et al.^27^	SYNTAXES. BEST, FREEDOM, EXCEL	7362	Patients with complex coronary artery disease	SYNTAX score II 2020	5-year MACE (all-cause death, non-fatal stroke or non-fatal myocardial infarction) and 10-year all-cause deaths	Yes
17	Sen et al.^28^	COMPASS	18 278	Established at baseline and included into the study	CHA2DS2-VASc and CHADS2	36-month MACE follow-up (thromboembolic disease and arrhythmias)	Yes
18	Takahashi et al.^29^	BEST, EXCEL, FREEDOM	7 362	CABG vs PCI based on eight patient characteristics and smoking-treatment interaction	SYNTAX	5-year MACE follow-up	Yes
19	Clinical trial iCorMicA 2022^30^	iCorMicA	N/A (enrolling currently)	Enrolment based on scores from the questionnaire	SAQ, quality of life score	Planned: 10-year MACE follow-up (death, MI, heart failure, stroke/TIA, unstable angina and coronary revascularisation)	No

Note: Please see the full reference list of the article, Premsagar P, Aldous C, Esterhuizen T. Cardiac scoring systems, coronary artery disease and major adverse cardiovascular events: A scoping review. S Afr Fam Pract. 2023;65(1), a5683. https://doi.org/10.4102/safp.v65i1.5683, for more information.

ACUITY, acute catheterisation and urgent intervention triage strategY (NCT00093158); ACS, acute coronary syndrome; ASCVD, atherosclerotic cardiovascular disease risk score; BEST, bypass surgery and everolimus-eluting stent implantation in the treatment of patients with multivessel coronary artery disease (NCT00997828); CABG, coronary artery bypass graft; CASS, coronary artery scoring system; HADS2 and CHA2DS2-VASc, congestive heart failure, hypertension, Age ≥75 years (doubled), diabetes mellitus, stroke (doubled), vascular disease, age 65–74 years, sex category; COMPASS, cardiovascular outcomes for people using anticoagulation strategies (NCT01776424); CvLPRIT, randomised trial of complete versus lesion-only revascularisation in patients undergoing primary percutaneous coronary intervention for STEMI and multivessel disease; EXCEL, to establish the safety and efficacy of the commercially approved XIENCE family stent system (NCT01205776); HEART, history, ECG, age, risk factors, troponins; FREEDOM, future revascularisation evaluation in patients with diabetes mellitus (NCT00086450); GRACE, global registry of acute coronary events; ICorMicA, international study of coronary microvascular angina (NCT04674449); non-IRA, non-infarct-related artery; NSTEMI/STEMI/MI, non-ST elevation myocardial infarction/ST elevation myocardial infarction/myocardial infarction; NYHA, New York Heart Association; JBS3, Joint British Societies’ consensus recommendations for the prevention of cardiovascular disease risk score; PCI, percutaneous intervention; PROMISE, prospective multicentre imaging study for evaluation of chest pain (NCT01174550); SAQ, Seattle Angina Questionnaire; SYNTAX (SYNTAXES), synergy between percutaneous coronary intervention (PCI) with Taxus and coronary artery bypass surgery (NCT00114972) (the SYNTAX extended survival); TIA, transient ischaemic attack; TIMI, thrombolysis in myocardial infarction.

**TABLE 6:** Individual sources of evidence (*n* = 13).

No.	References	*N*	Group 1/treatment	Group 2/control	Group 3	Group 4	Hazard ratio/AUC†	95% CI†	*p* †
1	Palmerini et al.^12^	2627	854	825	948	-	HR 1.04	1.01–1.07	0.005
2	Genereux et al.^13^	2686	1084	523	578	501	HR 1.05	1.02–1.09	0.006
3	Dangas et al.^14^	1850	956	894	-	-	HR 1.35	1.06–1.73	0.014
4	Gershlick et al.^15^	296	150	146	-	-	HR 0.45	0.24–0.84	0.009
6	Taşolar et al.^17^	252	131	79	43	3	AUC 0.804	CI 0.750–0.851	0.001
9	Hirji et al.^20^	664	-	-	-	-	C-index 0.67	-	-
10	Yudi et al.^21^	1590	1050	900	-	-	HR 1.04	0.81–1.32	0.78
12	Redfors et al.^23^	457	155	150	152	-	HR 1.03	0.99–1.06	0.01
13	Ferencik et al.^24^	4415	3739	676	-	-	HR 2.73	1.89–3.93	-
15	Hara et al.^26^	7362	4318	3044	-	-	C-index 0.66	0.65–0.67	-
16	Takahashi et al.^27^	5180	1800	3380	-	-	C-index 0·65	0.61–0.69	-
17	Sen et al.^28^	18 278	9152	9126	-	-	HR 0.67	0.53–0.86	0.0012
18	Takahashi et al.^29^	7362	-	-	-	-	C-index 0.69	-	0.021

Note: Please see the full reference list of the article, Premsagar P, Aldous C, Esterhuizen T. Cardiac scoring systems, coronary artery disease and major adverse cardiovascular events: A scoping review. S Afr Fam Pract. 2023;65(1), a5683. https://doi.org/10.4102/safp.v65i1.5683, for more information.

AUC, area under the curve; HR, hazard ratio; CI, confidence interval.

† , For uniformity, the HR/AUC given is for all-cause MACE mortality/morbidity – for other MACE events (such as myocardial infarction), see Table 4.

## Discussion

This scoping review mapped the literature on the use of cardiac scoring systems to predict CAD and MACE using the search terms, ‘coronary artery disease’ and ‘cardiac scoring systems’ and ‘major adverse cardiovascular events’ in a Boolean search on PubMed, ScienceDirect, MedLine and Cochrane Library. There were initially 111 results found, and 92 were removed on application of inclusion and exclusion criteria. There were 19 final results obtained which can be divided into two dichotomous groups: six that had no participants and 13 that had participants. The six results without participants were clinical guidelines, a review article and one actively enrolling clinical trial. The focus was primary prevention which involved screening and detecting CAD using cardiovascular risk estimation scoring systems. Management options and the risk of MACE were briefly discussed but scored.

The remaining 13 research articles included participants with established CAD. At baseline, they were grouped according to their disease profile and risk factors. The scoring systems were directed to secondary and tertiary prevention in patients with established CAD at baseline and to predict the risk of future MACE up to 5 years ahead. These results were mostly published from completed landmark clinical trials. This added to the robust nature of these results.

All studies concurred that scoring systems were an objective, standardised and accurate method to predict disease risk and compare differences between groups of participants with an outcome of interest. Some studies^26,27,29^ have also demonstrated that scoring systems are constantly being revised and updated as newer research becomes available. Overall, the 19 studies identified highlight the concern for CAD and MACE and the need to objectively mitigate this risk. There is, however, limited research in this area, given that CAD remains a leading cause of death worldwide.^1^

### Limitations

The limitations of this method were as follows:

- Studies that were non-English without a complete translation were excluded.
- There were four databases used, and many more do exist that may hold more reliable results.
- Unlike a systematic review, this scoping review had a limited appraisal of the quality of the studies.
- The search did not extend to contacting authors or searching by open researcher and contributor identification (ORCID).
- A limited number of databases were searched, and further searches may find more results.
- Grey literature, non-English literature and literature before the year 2000 were removed.
- Note: If the Boolean search included only ‘cardiac scoring systems’ and ‘coronary artery disease’ (leaving out ‘major adverse cardiovascular events’), just the two search terms would have found a *great abundance* of results on primary prevention of CAD using cardiovascular risk estimation scoring systems. The addition of the third search term, ‘major adverse cardiovascular events’, filtered the potentially abundant results to only those six that discuss MACE, even though no scoring systems for MACE are provided.

### Further recommendations

Further interrogation of the quality of the evidence in the studies found is necessary. A systemic review may be undertaken.

## Conclusion

This scoping review demonstrates a small volume of literature on using cardiac scoring systems to screen CAD (primary prevention) and/or prevent or treat MACE (secondary prevention). However, these 19 results were comprehensive because they included a wide spectrum of research, from guidelines for suspected chest pain patients to research predicting all-cause morbidity and mortality from MACE in 5 years. There is, however, sparse research when all three Boolean terms, ‘cardiac scoring systems’ and ‘coronary artery disease’ and ‘major adverse cardiovascular events’ are used.

While there is a paucity of research on the searched question, the desired number of results remains arbitrary because of the quality of the literature. Nonetheless, the gathered evidence suggests that scoring systems remain objective methods to help establish a diagnosis of CAD (cardiovascular risk estimation scoring systems for primary prevention) or predict and intervene CAD or MACE (secondary prevention). This applies specifically to areas of clinical uncertainty or to standardise patient results for comparison in research.
